# Prevention perspective in orthodontics and dento–facial orthopedics

**Published:** 2008-11-15

**Authors:** E Ionescu, E Teodorescu, A Badarau, R Grigore, M Popa

**Affiliations:** * University of Medicine and Pharmacy ‘Carol Davila’ Bucharest, Faculty of Dental Medicine, Department of Orthodontics and Dentofacial OrthopedicsRomania; ** University of Medicine and Pharmacy ‘Carol Davila’ Bucharest, Faculty of Medicine, Department of Physiology Romania; *** University of Medicine and Pharmacy ‘Victor Babes’, Timisoara, Faculty of Dental Medicine, Department of Orthodontics and PedodonticsRomania

## Abstract

In the present context of the public health directions, considering WHO main objective, that ‘all people of the world could reach the highest possible health level’, in medicine, the accent is put on prevention. In spite of the important progresses achieved in orthodontics field, the treatment still remains a symptomatic one. In this context, we must ask ourselves what are the prevention theoretical and practical coordinates in orthodontics, which measures are available or could be elaborated for preventing the malocclusions development. From the clinical point of view, the most important element of the new perspective is that most of the cases of anomalies which in the present are cured by orthodontics are induced by functional and environmental factors and they can theoretically be prevented. Thus, the identification, control and guidance of the environmental factors which adjust the growing of the maxillaries and of the other cranio-facial structures would be the main target of a prevention program in orthodontics.

## Introduction

The clinical and public health research have proved that a number of individual, professional and community prevention measures are efficient in preventing most of the oral diseases. The optimum intervention is not universally valid and possible due to the costs or the limited resources existing in certain communities or countries. This and the emphasis on primary prevention of oral pathology, represent a challenge for many countries, in particular for those who are under development and those disposing of a transition economy and health system.

The greatest challenge of the future will be to turn knowledge and expertise acquired in preventing diseases into active programs. The opportunities are represented by the extension of prevention and promotion of oral health to the public, by means of community programs aiming at informing the community and benefiting from the disease prevention measures. 

In 2004, WHO published the *Global Program for Oral Health Promotion* in view of improving oral health during the 19^th^ century, program that emphasizes the fact that despite the great oral health improvement of the entire world population, the problems still persist, particularly for the disadvantaged groups. This strategy primarily takes into consideration the diseases determined by the common risk factors, which can be prevented, represented by life style, diet, hygiene, vicious habits etc. (6).

In this respect, FDI and OMS drafted the objectives for 2020:

- Mitigating the impact of oral contamination, malocclusions and craniofacial anomalies on psycho– social health and development.
- Mitigating the impact of oral and craniofacial manifestations of the systemic diseases and the use of these manifestations for the precocious diagnosis, the prevention and the efficient management of the systemic diseases.

In dental medicine, the cariology may be considered the forerunner in prevention while other disciplines, orthodontics included, keep on being interested in symptomatic therapy. Even though functional and orthopedic therapy has been given more importance, in orthodontic therapy, the accent is put on malpositioned teeth alignment, by their mechanic movements. It's difficult to understand how the more the teeth movement mechanisms improved, the less interest for the etiological and anomaly development aspects is showed. We ask ourselves what are the theoretic and practical coordinates in prevention in orthodontics, what are the available measures or what could be elaborated in order to prevent the development of malocclusions. It will be interesting to know which measures can be used in the interceptive or corrective therapy for stopping the undesirable development in an incipient stage and for assuring a later normal development of the dento–facial complex. The importance of the questions on the precocious treatment has been analyzed, from the economic efficiency point of view, by Pulkinen and Pulli (1991) and later by Curzon, cited by Varrela and Alanen (5), who has emphasized the necessity of comparing corrective or interceptive interventions with a traditional orthodontic type of therapy in a cost–efficiency analysis. The observations on prevention potential in orthodontics are based on practical experience more than on controlled studies. Nowadays, large clinical studies are achieved in Finland, aiming at estimating the precocious orthodontic treatment efficiency and results. The major principles of treatment strategy applied in study are diagnosing the anomaly in the decidual dentition at the age of 5 or 6 years, initiating the treatment after the emergence of the first permanent molars or in some cases even earlier and directing the growing of the jaws. The strategy aim is to correct, in early mix dentition, the disturbances which have occurred or are about to appear and the precocious treatment argument is to obtain a maximum benefit from the high plasticity of the facial bones of the younger children. Lately, both craniofacial biology and clinical orthodontics have known an accelerated development, which justify a new evaluation of the prevention in orthodontics. 

## Considerations on malocclusions ethiopatogeny

In order to evaluate the perspective and the potential of prevention in orthodontics it is important to study the malocclusions ethiopatogeny. The knowledge on malocclusions ethiopatogeny is even now insufficient, many questions still having no answers.

Thus, for a long period of time, genetics has been primarily involved in malocclusions. In this respect, the development of the occlusion has been considered as following a coded genetic program and the anomaly has been perceived as result of a non–balanced growth of the craniofacial structures due to an unfortunate genes combination. 

This point of view that influenced both practical clinic and the orthodontics research is first of all based on the results of the studies on twins and the family problems. Classical genetic methods that have been involved in studies on twins and family overestimated the importance of the genetic factors, mainly due to the failure of these methods to separate the environmental effects or the environment–genes interaction of the proper genetic influences. A reasonable separation of the genetic and environment effects was made possible after 1970, when new genetic methods had been developed. Ever since, craniofacial studies revealed that the role of the genetic factors in controlling the development of the occlusion and the anomalies is less important than it was believed. The results of several studies on twins pointed out that, although it had been found significant genetic variations in several occlusion characteristics, they do not hereditary transmit but in a percentage of 25-30%. Moreover, a longitudinal analysis on blood relatives (brothers), concluded that ‘most of the occlusal modifications are more likely acquired than inherited’ (3,4). By Proffit, the main types of dento–maxillary anomalies are related to the abnormal growth of the alveolar processes, of basal parts of the jaws or a combination thereof (4). Without omitting or minimizing the role of the genetic factor, the observations of this study show that a large part of the malocclusions are determined by environmental factors. That is why this part of the modifications may be influenced by the preventive procedures.

In order to explain the modifications which appear under the influence of environmental factors, three main hypotheses/directions have been promoted, known as **functional hypotheses**. First of these theories incriminates the modifications of the diet and the alteration of masticatory activity, the second incriminates the factors which concur to the disturbance of the other functions which involve the dento–maxillary apparatus (breathing, deglutition, speaking) and the third which incriminates the vicious habits.

### The diet and the masticatory activity modifications

The industrial revolution induced an important adjustment of the energy content particularly of diet consistency. This adjustment led to the decreasing of the mastication need, to the alteration of maxillaries growth and to the increasing of anomalies frequency (1, 2). This means that the activity level of masticatory muscles is an important controller of the jaws growing. Experimental studies showed that animals which were bred with a low consistency food instead of high consistency natural diet, have a minimum masticatory function, smaller jaws and they develop the same anomalies observed to men. Moreover, histological studies showed that the activity level of the masticatory muscles (adjusts the cranial and facial bones development, influencing not only the sutures growing but also the bone apposition and resorption (3,5).

During masticatory activity, the jaws are receiving, beside the teeth pressures, a direct pressure born from the strong contractions of the tongue, lips and cheeks. The intense muscular activity is associated with a higher sanguine intake, which assure to the maxillary bones better development conditions.

### The modifications of the functions which implicate the dento–facial complex (breathing, deglutition)

Besides the masticatory muscles, to the craniofacial growing a great importance have the muscles which are maintaining the head posture or those participating to the other functions of dento–maxillary apparatus, such as breath, deglutition and speaking The perturbation of one of the function of the dento–facial complex frequently attracts the perturbation of the others, considering the close relation of interdependence and interconditioning, existing among them.

**Fig. 1:**
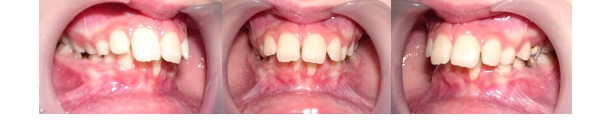
Patient with maxillary compression as a result of oral breathing and gingival retraction at left lower incisor

The association of respiratory and dento–maxillary disturbances was first observed by Robert in 1943. Ever since, several experimental research and clinical observations (Robin, Bimler, Gudin, Muller, Schwarz, etc) proved that there are obvious correlations between the respiratory and the dento–maxillary anomalies (3,4,5). Considering that, it is accepted that the increasing of the allergy frequency or higher activity of another factor which alters the functional dimension of the nasopharyngeal cavity may be correlated with a higher frequency of the specific dento–maxillary anomalies. Today it is known that oral breathing and head extension could affect the maxillaries growing, concurring to the dento–maxillary anomalies–maxillary compression.(Class 2/1)(Fig. 1).

Revealing the oral breathing on its debut can prevent with real results the evolution toward pathologically of jaws development and implicitly of jaws relations, therefore the settlement of malocclusions. This can be achieved by an ORL examination which will establish not only the possible obstacles existing in the nasopharyngeal passage but also the muscular behavior, mostly of the orbicular muscles. Stopping the evolution toward malocclusions can be achieved by the reeducation of the respiratory function (in subjective oral breathing), or by a surgical intervention of removing the possible obstacles existing in the superior respiratory airways, obligatory followed by functional reeducation (in objective oral breathing). The breathing reeducation exercises, after the removal of adenoid processes are considered to be of great importance even in the case of children who are not presenting malocclusions.

The problem debating the deglutition influence in the dento-facial development was raised much later, in 1946, when the orthodontists realized that ‘there patients had also a tongue’ (Graber) (3).

**Fig. 2:**
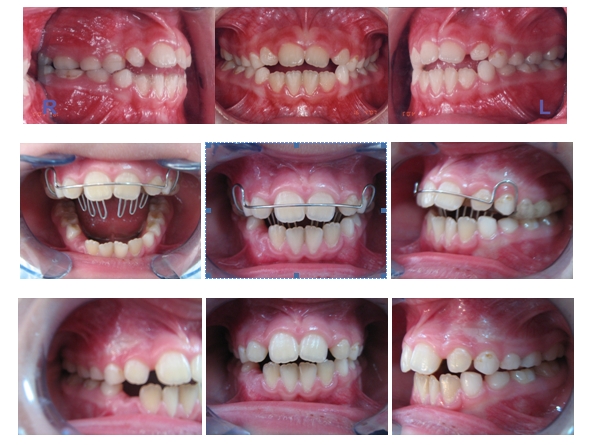
Pacient with anterior open byte consecutive to the tongue thrust in treatment (intraoral aspect– after 1 year of treatment)

**Fig. 3:**
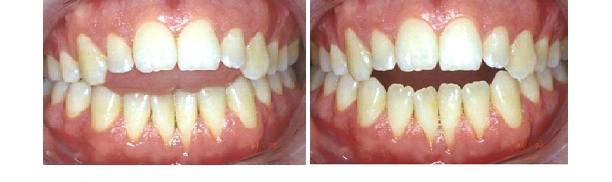
Pacient with anterior open byte consecutive to the tongue thrust in treatment (intraoral aspect– after 1 year of treatment)

The deglutition is another function which implicates the components of the dento–maxillary complex, the most important being the tongue. The deglutition, in a physiologically evolution has three stages, dependent and in correlation with the teeth eruption and the jaws relation development (infantile deglutition–before the deciduous teeth eruption, the transition deglutition– after the eruption of the incisors, the adult deglutition– after the dental arches are constituted). Most frequently the alteration of this function is identified as infantile deglutition after the age of 2Ɖ–3 years (the tongue interposition between the dental arches) which on dento–facial complex has consequences like the dysfunctional open byte syndrome (by tongue thrust). In the matter of the patients who are presenting malocclusions determined by these disturbances, the therapeutic efforts must be centered on the modification of deglutition comportment. The reeducation of deglutition can be achieved by exercises or by tongue habit appliances, orthodontic appliances which prevent the tongue thrust. These appliances have a double role, to remove the abnormal tongue forces and to reeducate the function. We present the case of a 9 years patient, with dysfunctional anterior open byte caused by tongue thrust. One year of wearing orthodontic appliances made possible a further vertical eruption of the teeth and a considerable decreasing of the vertical inocclusion space (Fig. 2,Fig. 3).

In the next figure we present by antithesis a 30 years patient with the same type of open byte (caused by tongue thrust) who didn't beneficiate of functional reeducation. There are notable the malocclusion consequences caused by the hypofunction of the anterior teeth, respective the periodontal disorder, particularly noticeable in the lower arch (severe gingival retractions) 

### Vicious habits

**Fig. 4:**
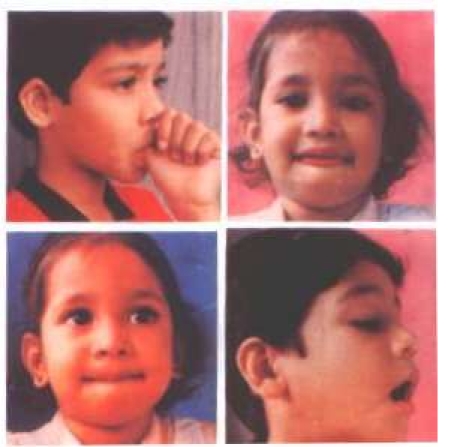
Patients presenting different types of vicious habits

An analysis of the factors that may interfere with the balance of the oro–facial equilibrium forces in the development of the dento–facial complex cannot exempt a group of influences, highly discussed during the malocclusions ethiopatogeny, known under the generic term of vicious habits. These are habitual actions, gestures achieved voluntarily and spontaneous by the child, practiced with a certain intensity and frequency, on a longer period of time, actions that during the development of the dento–facial complex can determine the emergence of malocclusions. Fig. 4

It is well known that the group of muscles which are influencing the facial growth and which are in straight correlation with the development of jaws and occlusion is made up of the facial musculature and by tongue muscles. The pressures developed by cheeks and lips from outside and by the tongue from inside, are representing important factors which are not only guiding the development of the occlusion but also are influencing the maxillary growth (4). If the balance between the cheek and lips muscles on one part and the tongue muscles by the other part is disturbed, it will be a high probability for skeletal and occlusion disorders to occur. In this respect, the vicious habits, particularly those of sucking and interposition representing a part of the modern lifestyle which is reflected by the decrease of breast feeding or other changes of the growing habits of the child, cause malocclusion, concurring to a higher frequency thereof.

We consider that from all the etiological factors involved in malocclusions, the prevention could be of great importance in correcting the vicious habits, but only if this type of habits and their consequences are known.

From the above mentioned facts it is obvious that the adjustment of the cranio–facial growth is under a less strict genetic control; on the other hand, it seems to highly depend on the influence of several oro–facial functions, especially during the post birth period. The factors related to masticatory, respiratory, vicious habits modifications or combination thereof are responsible with the emergence and the increase of the malocclusions. Clinically, the most important element of the new approach resides in the fact that, most of the anomalies that orthodontics treats today are induced by environment and functional factors that could, at least theoretically, be prevented. Thus, *the identification, control, and conduct/guidance of the environment factors that regulate the maxillaries and other craniofacial factors growth would represent the main targets of several approaches to set up a prevention and interception program in orthodontics*(2).

Prevention could be thus considered a possible alternative to the active orthodontic treatment. The preventive measures should target the provision of the normal function of the oro–facial muscles during the craniofacial growth process, thing that would lead to both the decrease of the number of malocclusion and the amelioration of the clinical aspects thereof for the benefit of health.
